# Life Cycle-Dependent Cytoskeletal Modifications in *Plasmodium falciparum* Infected Erythrocytes

**DOI:** 10.1371/journal.pone.0061170

**Published:** 2013-04-09

**Authors:** Hui Shi, Zhuo Liu, Ang Li, Jing Yin, Alvin G. L. Chong, Kevin S. W. Tan, Yong Zhang, Chwee Teck Lim

**Affiliations:** 1 Nano Biomechanics Laboratory, Department of Bioengineering, National University of Singapore, Singapore, Singapore; 2 Infrastructure System Laboratory, Department of Civil Engineering, National University of Singapore, Singapore, Singapore; 3 Singapore-MIT Alliance (SMA), National University of Singapore, Singapore, Singapore; 4 Laboratory of Molecular and Cellular Parasitology, Yong Loo Lin School of Medicine, National University of Singapore, Singapore, Singapore; 5 Department of Mechanical Engineering, National University of Singapore, Singapore, Singapore; 6 Mechanobiology Institute, National University of Singapore, Singapore, Singapore; University of Heidelberg Medical School, Germany

## Abstract

*Plasmodium falciparum* infection of human erythrocytes is known to result in the modification of the host cell cytoskeleton by parasite-coded proteins. However, such modifications and corresponding implications in malaria pathogenesis have not been fully explored. Here, we probed the gradual modification of infected erythrocyte cytoskeleton with advancing stages of infection using atomic force microscopy (AFM). We reported a novel strategy to derive accurate and quantitative information on the knob structures and their connections with the spectrin network by performing AFM–based imaging analysis of the cytoplasmic surface of infected erythrocytes. Significant changes on the red cell cytoskeleton were observed from the expansion of spectrin network mesh size, extension of spectrin tetramers and the decrease of spectrin abundance with advancing stages of infection. The spectrin network appeared to aggregate around knobs but also appeared sparser at non-knob areas as the parasite matured. This dramatic modification of the erythrocyte skeleton during the advancing stage of malaria infection could contribute to the loss of deformability of the infected erythrocyte.

## Introduction

*Plasmodium (P.) falciparum* causes the most virulent form of human malaria, which attributes to repeated life cycles of growth of the parasite in the erythrocyte. During growth, the parasite extensively modifies the membrane of the host cell, resulting in changes in morphology, deformability and adhesive properties of the host erythrocyte [1]. The erythrocytes become stiffer after infection, generally reflecting changes in the structure of the membrane cytoskeleton [2], [3]. One of the most striking structural alterations on the membrane of the host cell is the formation of knobs, which are composed of parasite-expressed proteins, such as *P. falciparum* erythrocyte membrane protein 1 (PfEMP1) and knob-associated histidine-rich protein (KAHRP) among others [2]. These knobs interact with the spectrin network via the attachment of KAHRP to the spectrin-actin-protein 4.1 junction [4] or direct binding of KAHRP to spectrin tetramers [5]. Such interaction of knob proteins with spectrin-based cytoskeleton has been proposed to partially contribute to increased membrane rigidity and altered morphology of infected erythrocytes [6]. Besides, malarial parasite infection could also induce the rearrangement of cytoskeletal proteins, especially spectrins, the major determinants of shear elasticity [7]. In fact, the host cell cytoskeletal proteins are vulnerable to being fragmented by parasite proteases plasmepin-2 [8], falcipain-2 [9], [10], or others [11] during the maturation of parasite, and host cell calpains at the schizont stage [12]. However, the changes in host cell cytoskeleton caused by *P. falciparum* infection and development have not been well quantitatively elucidated.

In imaging the fine structure of erythrocyte cytoskeleton, atomic force microscope (AFM), has advantages over electron microscopy in terms of ease in sample preparation and minimal specimen perturbation [13], [14], [15]. AFM imaging from the extracellular surface unveiled that the erythrocyte cytoskeleton becomes less dense after malarial parasite infection [16], [17]. However, AFM imaging from the extracellular surface can only detect 80% of the spectrins [13] and has difficulty examining the connections between knobs and cytoskeleton or the detailed changes of the spectrin network [17]. In contrast, imaging from the cytoplasmic surface exhibited the real morphology of the spectrin network [13], and the native state of the cytoskeleton can be maximally preserved via the tight binding of erythrocytes to substrates [14]. Indeed, we can very clearly observe not only the knob structure and the connections between knobs and the spectrin network but also the distortion of the spectrin network at the schizont stage [17]. To more robustly characterize the stage-related alteration of *P. falciparum* infected erythrocyte cytoskeleton, we present quantitative measurements of the structural changes of infected cell cytoskeleton at the various stages of infection in terms of spectrin network mesh size, spectrin protein length and spectrin abundance. We demonstrate that the host spectrin network is gradually rearranged upon infection and development of the parasites, and provide detailed insights that are substantial in terms of understanding the temporal nature of these events.

## Results

**Figure 1:**
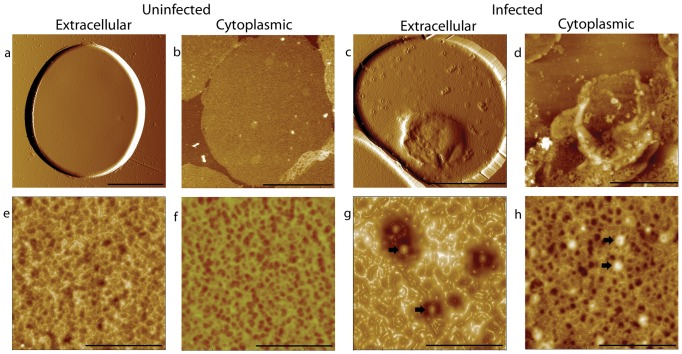
AFM images of the outer and inner surface of uninfected and infected erythrocytes. (a) Intact uninfected erythrocyte; (b) cytoplasmic-surface-exposed uninfected erythrocyte membrane (one layer); (c) Intact trophozoite stage infected erythrocytes; (d) cytoplasmic-surface-exposed trophozoite stage infected erythrocyte membrane (one layer); (e-f) Plot view of high magnification of (e) uninfected erythrocyte membrane outer surface, (f) uninfected erythrocyte membrane inner surface, (g) infected erythrocyte membrane outer surface and (h) infected erythrocyte membrane inner surface. Knobs were indicated by black arrows. Bars: 4 µm (a-d); 500 nm (e-h).

AFM imaging of the extracellular surface of normal erythrocytes revealed the cytoskeletal network underlying the membrane (Fig. 1a, e). However, the cytoskeleton observed from the outer surface may not represent the full details of the spectrin network, as there are also membrane proteins, glycocalyx and the phospholipid bilayer which may block the complete information on the structure of the cytoskeletal network. Similarly, trophozoite stage infected erythrocytes were also investigated from the extracellular surface by AFM and the structure and the distribution of knobs were easily observed (Fig. 1c, g) [18]. Although the spectrin network looked sparser than uninfected erythrocyte (Fig. 1e, g), the connection between knobs and cytoskeleton and the detailed changes of the spectrin network were hard to detect from the outer surface. As for the schizont stage, it is even harder to image the cytoskeleton from the outer surface, because the parasites occupy a majority part of the host cell and the outer surface becomes more rough and undulating [18]. Consequently, we turned to observing the cytoskeletons directly from the cytoplasmic surface.

**Figure 2:**
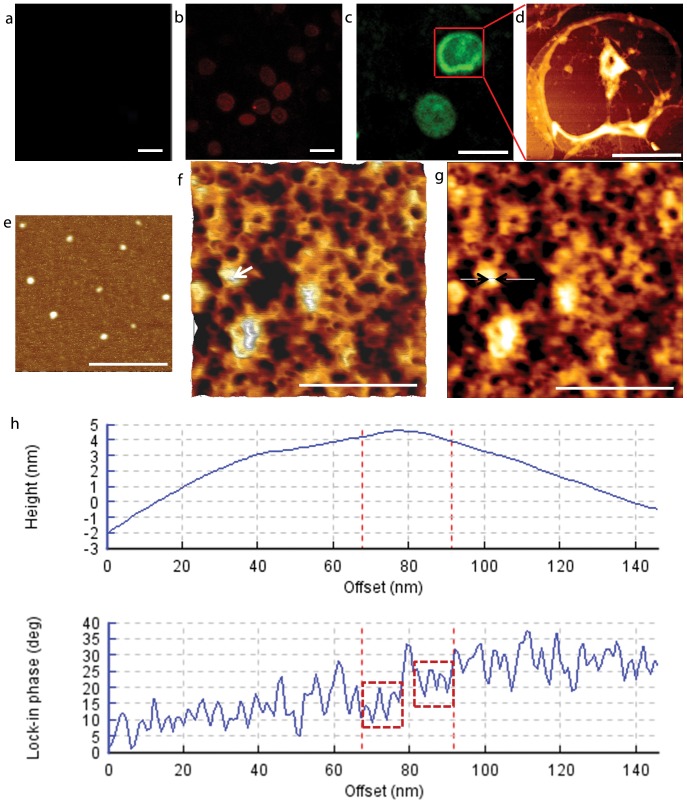
QDs targeting the cytoplasmic surface of infected erythrocyte membrane proteins. (a) No primary antibody and QDs only for control. (b) Mouse anti-spectrin followed by goat anti-mouse Fc-conjugated QD605; (c) Rabbit anti-KAHRP followed by goat anti-rabbit Fc-conjugated QD525; (d) Interested area from (c) was imaged using AFM; (e) AFM image of QD525 on cover slips; (f) Plot view of high magnification of QDs targeted KHARP proteins (zoomed from d), a QD is indicated by a white arrow; (g) AFM height image of the same area as (f), a short section of 79.34 nm through the QD was indicated by a white line with two black head to head arrowheads; (h) The height and the lock-in phase plot of the section draw in (f), the QD was verified by the pattern of the phase plot between the two red dash lines, where the negative shifts at the peripheral part of the QD indicated by two dashed red boxes, confirmed that this particle was a QD [37]. Bars: 10 µm (a-c); 4 µm (d); 400 nm (e); 500 nm (f & g).

**Figure 3:**
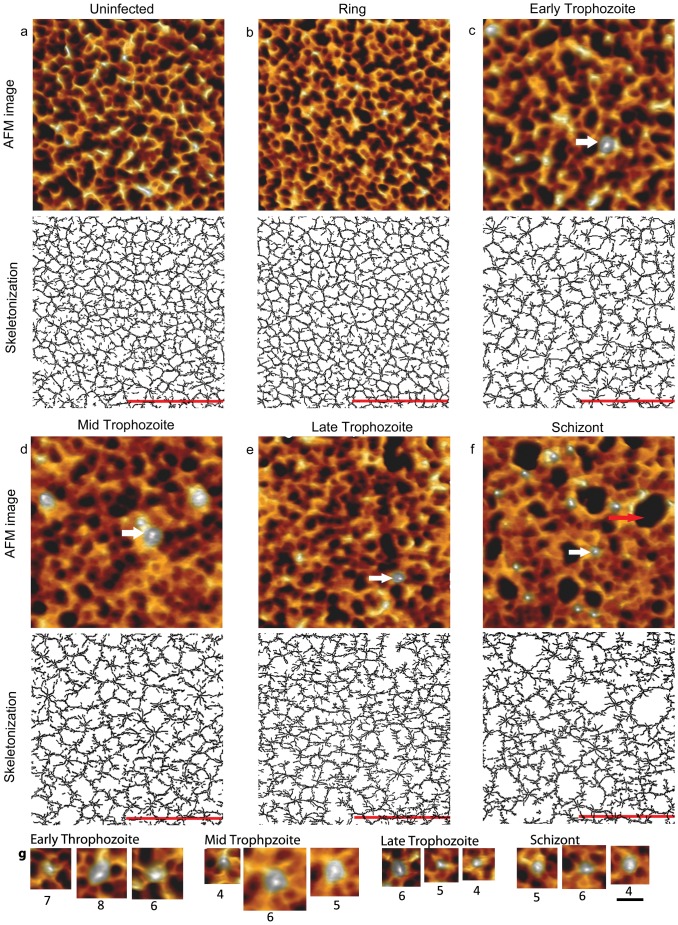
Erythrocyte cytoskeleton alteration after *P. falciparum* infection. (a-f) 3-D view of the high resolution AFM images and corresponding skeletonized cytoskeleton: (a) Uninfected erythrocyte; (b) ring; (c) early trophozoite; (d) mid trophozoite; (e) late trophozoite and (f) schizont infected erythrocyte (inner surface). Knobs and huge meshes are indicated by white and red arrows respectively. (g) Knob structures and their connections to host cell spectrin networks with labels of number of filaments connected to the knob at different stages. Bars, 500 nm (a-f); 100 nm (g).

The exposed cytoplasmic sides of the membranes of both the uninfected (Fig. 1b) and infected (Fig. 1d) erythrocyte were approximately 7.5–8.5 µm in diameter and the thickness of the membranes ranged from 5–10 nm except at the knob areas. Comparing to the finely weaved spectrin network of uninfected erythrocytes (Fig. 1f) [13], [14], [15] where spectrins were identified by gold particles previously [13], protrusions with 60 to 120 nm in diameter and 7 to 20 nm in height were formed after infection (Fig. 1g, h) [17]. These protrusions, identified as knobs using quantum dots (QDs) (Fig. 2), are connected to the host cell cytoskeleton via the binding of KAHRP to the spectrin-actin-protein 4.1 junction [4] or to spectrin tetramers [5] and thus resulted in four to eight filaments (spectrin tetramers) extending out from each knob (Fig. 3g). The density of knobs increased to 7±2/ µm^2^ until the schizont stage as previously reported [18]. Also, the spectrin network of infected cell appeared unevenly distributed, suggesting the feasibility of using AFM to explore the process of the modification of infected cell cytoskeleton from the cytoplasmic surface.

**Figure 4:**
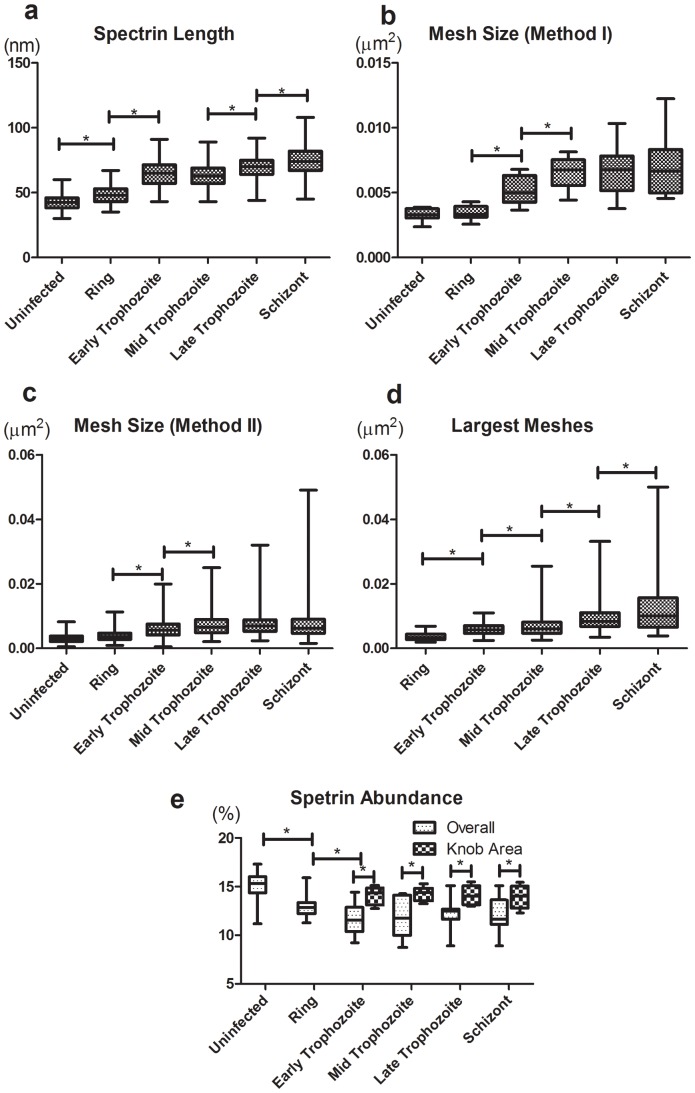
Statistical analysis of the spectrin networks at different infection stages. (a) The spectrin length (n = 100, 100, 170, 110, 120, 110 for uninfected, ring, early trophozoite, mid trophozoite, late trophozoite and schizont respectively); (b) The mesh size (M*_i_*) using Method I at different stages (n = 9, 10, 19, 11, 30, 10 for uninfected, ring, early trophozoite, mid trophozoite, late trophozoite and schizont respectively); (c) The mesh sizes using Method II; (d) The largest mesh sizes (n = 100 for all the stages); (e) Spectrin abundance (overall and at knob areas) (Csn) (n = 10, 10, 15, 15, 17, 9 for uninfected, ring, early trophozoite, mid trophozoite, late trophozoite and schizont respectively). * P<0.001; ** P<0.005; *** P<0.05 (ANOVA for a, b, c and e; Kolmogorov-Smirnov Test for d).

To characterize the changes of the spectrin network arising from parasite-mediated modification, we first purified infected erythrocyte at each specific stage, i.e. ring (∼10 hours post-infection (hpi)), early trophozoite (∼20 hpi), mid trophozoite (∼25 hpi), late trophozoite (∼30 hpi) and schizont stage (∼45 hpi) (see Methods and Fig. S1). This purification of infected erythrocytes at specific stages enabled us to statistically and quantitatively study the gradual changes of the spectrin network upon *P. falciparum* infection. The gradual changes of the spectrin network at different stages were then viewed by high-resolution AFM imaging of the cytoplasmic-surface-exposed cell samples, followed by skeletonization of the spectrin network using developed ridge detection (see methods) (Fig. 3a-f). As expected, knobs started to appear from the early trophozoite stage (Fig. 3c). Interestingly, the spectrin network around the knobs tended to become denser than non-knob areas as the parasites matured (Fig. 3c-g). In contrast, the spectrin network at non-knob areas appeared sparser with advancing stages of infection (Fig. 3c-f) and extraordinarily large meshes were formed at the schizont stage (Fig. 4f) [19], [20], suggesting degradation of spectrins or other cytoskeleton-related proteins which might facilitate the release of newly generated parasites from the host cells upon rupture. To quantify the gradual degradation of the cytoskeleton, we conducted statistical analysis on the spectrin lengths, mesh sizes and spectrin abundance measured.

### Spectrin lengths at non-knob areas

The randomly chosen spectrin filaments at non-knob areas at each specific stage appeared to follow normal distributions in length (Fig. S2). Notably, the spectrin tetramers measured from AFM images were much shorter than those measured from TEM images of extended spectrin network [21], [22], since the tight binding of the membranes to the PHA-E substrates maximally maintained the structure of the cytoskeleton *in situ*. The length of spectrin filaments in uninfected erythrocytes was 43±5 nm, which was consistent with previous findings [23]. Interestingly, the spectrin tetramers at the ring stage (∼10 hpi) started to elongate up to an average length of 48±7 nm compared to that of uninfected erythrocytes (P<0.001) (Fig. 4a), an increase by about 5 nm. The length of spectrin then increased to 64±9 nm at the early and middle trophozoite stage, about 33% longer than at rings (P<0.001), while there was no significant difference between the mid-stage trophozoites (∼25 hpi) and the early trophozoites (∼20 hpi) (Fig. 4a). Further, the spectrin tetramers were further elongated to 69±10 nm at the late trophozoite stage (∼30 hpi) (P<0.001) and finally to 75±11 nm at the schizont stage (∼45 hpi), which were longer than the spectrins of late trophozoites (P<0.001) (Fig. 4a). In total, the spectrin tetramers were gradually elongated by about 32 nm and more than 74% when compared between uninfected erythrocytes and schizont-infected erythrocytes (P<0.001) (Fig. 4a), which demonstrates the progressive extension of the spectrin tetramers during the maturation of the parasites inside the host cells.

### Spectrin mesh size (i.e., the size of a spectrin enclosed area)

We first used the method of measuring the average mesh size (Method I) to monitor the general changes of spectrin meshes during infection (see Methods). As shown in Fig. 4b, the average mesh size of the infected erythrocyte cytoskeleton at ring stage was 0.0035±0.0005 µm^2^, comparable to that of the uninfected erythrocytes. In contrast, the meshes started to expand at the early trophozoite stage to 0.0051±0.0011 µm^2^ and about 48% larger than the ring form meshes (P<0.001). The meshes then continued to increase by about 30% at the mid-trophozoite stage (P<0.001). Henceforth, no significant expansion was observed from the mid-trophozoite stage (0.0067±0.0011 µm^2^) to the late trophozoite stage (0.0066±0.0016 µm^2^) and to the schizont stage (0.0071±0.0025 µm^2^). Taken together, the spectrin network underwent more than two times expansion from the ring to the schizont stage (P<0.001) (Fig. 4b). To verify the accuracy of Method I, we also manually measured the mesh sizes at each infection stage (Method II) (Fig. 4c). The averages of the mesh sizes at each specific infection stage were consistent with the data obtained using Method I (Fig. 4b and 4c).

Unlike healthy erythrocyte cytoskeleton (Figs. 1f and 3a), the spectrin networks of infected cells, especially those at the late stages, were not uniformly distributed (Fig. 3b-f). Some super-enlarged meshes, which were substantially larger than the theoretically biggest circle enclosed by three fully extended spectrin tetramers (around 200 nm), i.e. 0.029 µm^2^, appeared at the schizont stage (Fig. 3f and 4c-d). In view of this, we paid special attention to those obviously enlarged meshes of the infected cell cytoskeletal network and used Method II to statistically study the enlarged meshes at each specific infection stage. The sizes of these enlarged meshes at each particular stage appeared to follow a log-normal distribution (see Fig. S3) and there was a general right-shift of the distribution of the largest mesh sizes from the ring stage to the schizont stage (see Fig. S3f). The largest mesh sizes at the ring stage were 0.0036±0.0011 µm^2^, however, for the early trophozoite stage, they were 0.0059±0.0018 µm^2^ or are about 63% larger (P<0.001) (Fig. 4d). The largest spectrin meshes of the middle trophozoites were about 26% bigger than those of the early trophozoites (P<0.05) (Fig. 4d). Also, the largest meshes of the late trophozoites expanded about 36% over those of the mid trophozoites (P<0.001) (Fig. 4d). Moreover, the sizes of largest spectrin meshes at the schizont stage increased by 26% from those of the late trophozoites (P<0.002) (Fig. 4d). In total, the largest meshes of the schizonts were about 257% larger than those of ringforms, implying that certain meshes were enlarged by over 3.5 times during the transition from ring to the schizont stage (P<0.001) (Fig. 4d).

In comparison, both of the results obtained from the two methods demonstrated that the spectrin network at non-knob areas became expanded as parasites developed inside the host cells, which was in agreement with the elongation of the spectrin filaments as the infection stage advances. However, the largest meshes differed more (at least 26%) between any two adjacent stages (Fig. 4d), while the changes in the average of the mesh size were limited at the later stages (see Fig. 4b, c). This suggests that some of the meshes had undergone dramatic expansion while others had little or no change.

### Spectrin abundance (ratio of cytoskeleton occupied areas to membrane area)

To determine whether the modifications of the spectrin network cause partial dissociation of the spectrin tetramers or the loss of cytoskeletal or cytoskeleton-associated proteins, we statistically studied the abundance the spectrin network (see Methods) at different stages of parasitic infection (Fig. 4e). The spectrin abundance of the infected cells at the ring stage was about 13% less than the ratio of the uninfected erythrocytes (P<0.01). The ratio then decreased at the early trophozoite stage to just about 90% of the ratio of the ringforms (P<0.05), whereas there was no significant difference from the early trophozoite to the schizont stage. In total, the spectrin abundance was reduced by about 20% from the uninfected to the schizont stage (P<0.001), implying substantial break-down of the host cytoskeleton arising from malarial infection.

**Figure 5:**
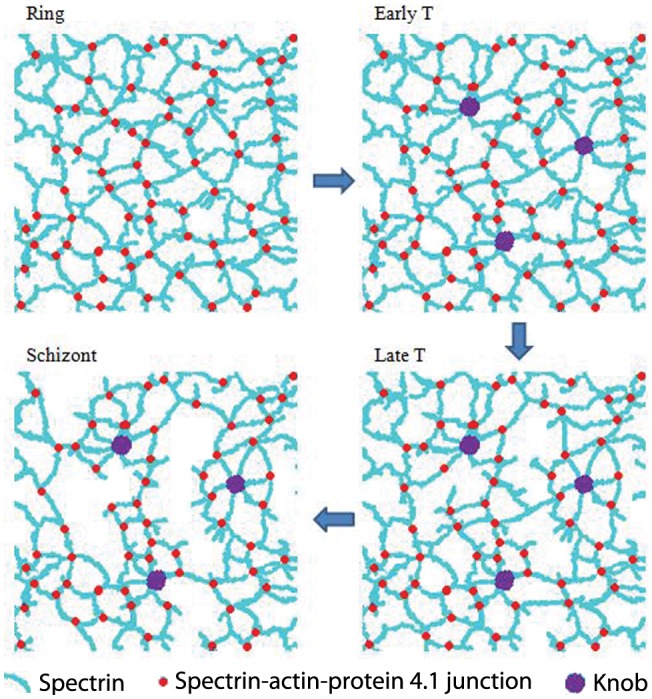
Schematic diagram of the alterations of cytoskeleton from the ring stage to the schizont stage. Knobs exist from the early trophozoite stage; spectrin network undergoes gradual distortion from ring to schizont stage; and huge meshes exist at the schizont stage.

The loss of spectrin abundance is in line with the elongation of the spectrin tetramer and the expansion of the spectrin mesh size at the ring and early trophozoite stages. However, while the spectrin mesh grew larger and the spectrin tetramers at non-knob areas appeared longer from the mid trophozoite to the schizont stage, the spectrin abundance seemed unchanged. A possible reason is that the spectrin network underwent complex molecular modifications resulting in the rearrangement of the spectrin proteins. That is, along with more knobs existing from early trophozoite stage to Schizont stage, more spectrin accumulated at the knob areas, as the spectrin abundance was higher at knob areas (Fig. 4e) but expanded or broke at the non-knob areas, while the number/abundance of the spectrin molecules remained relatively unchanged (Fig. 5).

## Discussion

Currently, very limited information is available that describe molecular-level physical modifications that happen to a human red blood cell upon infection and development of malarial parasites. Understanding changes that occur to cytoskeletal network integrity and its components can provide new information pertaining to the extent of host-pathogen interactions and can lend support to various therapeutic explorations targeting such changes. Our data derived from AFM analysis demonstrated that at the early stages of infection, the host spectrin began to elongate (Fig. 4a), subsequently the spectrin network started to become sparser (Figs 4b, c) and the overall spectrin abundance was decreased (Fig. 4e). These changes may be due to the initial export of parasite-expressed ring-infected erythrocyte surface antigen (RESA) proteins which bind to the host cell cytoskeleton [24] and significantly affect the deformability of host cells at the early ring stage [25]. As to the trophozoite and schizont stage, the cytoskeleton became even sparser and the spectrin tetramers appeared more extended (Figs. 4a-d), which could be contributed to the formation of knobs and the loss of erythrocyte membrane-adaptor proteins such as Adducin and others [20]. The spectrins also appeared to aggregate at knob areas (Figs. 3 and 4e), while the spectrin tetramers were elongated and the meshes were enlarged at non-knob areas. It is possible that during the formation of knobs, some of the spectrins may get pulled and relocated to the knob areas, thereby resulting in enlarged meshes and elongated spectrin tetramers at non-knob areas. Also, the formation of knobs disturbs the orderly distribution of the spectrin network. Parts of the spectrin tetramers may lose their freedom of the dynamic association-and-dissociation movement. Consequently, these parasite-exported proteins coupled with extended spectrin tetramers may have stiffened the host cell cytoskeleton. In fact, it has been demonstrated that the KAHRP and PfEMP3 proteins greatly contributed to the host cell rigidification [26]. Beside the formation of knobs, the cleavage of the spectrin proteins by parasite-expressed proteases [8], [10], [11] may result in the de-stabilization and collapse of the spectrin network. The cleavage of infected cell cytoskeletal proteins could affect spectrin anchoring to the erythrocyte membrane, interrupt spectrin-spectrin interaction and cause loss of certain cytoskeleton-related proteins; thereafter triggering the breakage of spectrin network, enlargement of the spectrin meshes and the reduction of the spectrin coverage ultimately leading to facile exit of merozoites at the end of an intra-erythrocytic cycle.

## Conclusions

The ability of AFM to image the cytoplasmic surface of malaria infected erythrocyte membrane has enabled us to systematically document the following alterations of the spectrin network from the ring to trophozoite and to the schizont stage: (1) formation of knobs on the host cell membrane at the early trophozoite stage; (2) partial stretching of the spectrin network at the non-knob areas from the early trophozoite stage; and (3) gradual increase in mesh size of the spectrin network during maturation of the parasites inside the infected cells. Furthermore, we also provided the first quantitative study of the gradual changes of the spectrin network of erythrocytes in terms of the mesh size, spectrin protein length and spectrin abundance during *P. falciparum* infection. These findings not only provide direct evidences of the gradual modification of the cytoskeleton of erythrocytes during malaria infection, but also yield insightful clues into the roles the parasite-exported proteins play in modifying the host cell cytoskeleton.

Finally, spectrin is the main cytoskeletal protein responsible for maintaining the shape and elasticity of the erythrocytes [7] and the deformability of the erythrocyte membrane partially relies on the regulation of spectrin tetramer association and dissociation [27], [28]. It is well known that the erythrocytes become stiffer [29] and less deformable [30] and more fragile [31] after *P. falciparum* infection. These gradual changes in mechanical properties of infected cells are now shown to be accompanied by the gradual changes of the cytoskeletal network as revealed by our AFM imaging. This study should offer some clues to the relationship between the mechanical property changes and the structural alteration observed in malaria infected erythrocytes.

## Materials and Methods

### Ethics statement

Neither human participants or animal work is involved in this research.

### Cell culture

*P*. *falciparum* parasites (3D7) were cultured in vitro using standard procedures [32] and gelatin flotation was carried out once a week to synchronize the culture as described previously [33]. Knobby infected cells were selected using platelets-coated glass Petri dishes as reported previously [17].

### Culture of 100% singular parasite infection

A simple method was used to obtain almost 100% pure single infection culture. Briefly, the subculture of the synchronized culture was conducted at the schizont stage and incubated in an incubator for about 1 h. The flasks were then capped tightly and put on an orbital shaker (Boeco) in a warm room (37°C). After that, cultures were rotated at 100 rpm for around 12 hours and put back into the incubator for normal culturing. This method is much more efficient than the method developed previously which can only reduce the multiple infection to around 7% [34].

### Cell sorting at different stages

MidiMACS separation set (Miltenyi Biotec) was used to enrich trophozoite and schizont stage infected cells [17]. Briefly, a LD column (Miltenyi Biotec) was inserted into a MACS separator and preload with 1 ml EDTA-BSA rinsing buffer (0.5% BSA (Miltenyi Biotec) and 0.2 mM EDTA (Miltenyi Biotec) in Phosphate buffered saline (PBS), pH 7.2). Meanwhile, 10 ml of parasite culture was pelleted by centrifuging and gently resuspended in 2 ml of rinsing buffer. The cell suspension was applied on to a preloaded LD column and rinsed with 2 ml rinsing buffer several times once the column reservoir was empty. The cells passing through the column were then collected (Portion I).When there was no red color solution seen passing through, 2 ml of rinsing solution was added and the column was taken out for collecting the effluent (Portion II). By using this method, the enriched late stage parasitized erythrocytes were around 98% in purity and the viability of parasites were not affected by the MACS enrichment procedure [35]. The two portions of cells collected from MACS separation were diluted with PBS to the concentration of 2-4×10^7^ cells/ml, and stained with 2.5 µg/ml Hoechst-33258 (Invitrogen) for 0.5 h in dark. The cells were then spun down and rinsed twice with PBS and resuspended in FACS sorting buffer (1 mM EDTA (Sigma), 25 mM HEPES (4-(2-hydroxyethyl)-1-piperazineethanesulfonic acid) (Sigma) in PBS, pH 7.4). After filtering through 40 µm cell strainer (BD biosciences), the cells were analyzed and sorted using FACSCalibur II (BD biosciences). FACS sorted infected cells at specific infection stages were all with singular parasite infection, and were of high purity (according to the Giemsa Staining results, see Fig. S1) and still intact and viable [36].

### Sample preparation

Thin smears of the culture were prepared for AFM imaging the outer surface of the uninfected and infected erythrocytes. Cytoplasmic-surface-exposed samples using the FACS sorted cells were prepared as described previously [17]. Briefly, (3-aminopropyl)triethoxysilane (APTES)-treated cover slips were treated with 150 µl of 1 mM Bis(sulfosuccimidyl) suberate (BS^3^) (Pierce) in PBS for 0.5 h and washed with PBS, followed by incubation with 150 µl of 0.1 mg/ml Erythroagglutinating phytohemagglutinin (PHA-E) (EY laboratories) solution for 2 h. The cover slips were rinsed with PBS three times and quenched with 0.1 M glycine for 15 min. After rinsing with PBS, the PHA-E coated cover slips were ready for use and can be stored in 4 °C for 2 weeks. The purified uninfected and infected cells at all different stages were incubated on the substrate for 3–4 h followed by rinsing with PBS. Then the cells were shear washed by 60 ml of 5P8-10 buffer (5 mM Na_2_HPO4/NaH_2_PO4, 10 mM NaCl, PH 8) using syringe at a angle about 20 degree. The cytoplasmic-surface-exposed-samples were either vacuum dried or stained with quantum dots (QDs) for AFM imaging.

### QDs staining

Some cytoplasmic-surface-exposed samples were blocked with 150 µl of 10% normal goat serum (Invitrogen) in 5P8-10 buffer for 1 h. The block solution was then poured off. The samples were incubated with 150 µl of primary antibodies (rabbit anti-KAHRP monoclonal antibody (gift from Dr Brain Cooke, Monash University), 1∶500, or mouse anti-spectrin polyclonal antibody (Sigma), 1∶500, or none for control) in 1% normal goat serum in 5P8-10 for 1 h. After rinsing with 5P8-10 for 3 times, the samples were incubated with 150 µl of anti-rabbit secondary antibody-conjugated QD525 (green color) or anti-mouse secondary antibody-conjugated QD605 (red color) (Invitrogen) respectively with the dilution of 1∶100 in 1% normal goat serum in 5P8-10 for 1 h. Afterwards, the samples were rinsed with 5P8-10 for 3 times, followed by a quick wash with distilled water, and then vacuum dried for AFM imaging. All the above incubations of the QDs staining were done in a humid chamber at room temperature. The optical or fluorescent images (2048×2048) were captured using the FV10-ASW 2.0 software (Olympus) and processed using the FV10-ASW 2.0 Viewer (Olympus,).

### AFM imaging of samples

Dimension 3100 AFM with a Nanoscope IIIa controller (Veeco) were used to image both the smeared samples and some of the cytoplasmic-surface exposed samples. Also, the NanoWizard II AFM (JPK Instruments AG) coupled with a confocal microscope (Olympus) were applied to image the cytoplasmic-surface-exposed samples which were used for statistical studies.

Model ORC8 probes with spring constant of 0.05 N/m (Veeco) were used to image the smeared cells in contact mode. Height and deflection images were obtained at a resolution of 512 × 512 pixels for 8 µm×8 µm or 1 µm×1 µm areas and scan rate of 0.5 to 2 Hz depending on the scan scale.

Super sharp probes, SSS-NCHR probes (Nanosensors) and ACTA-SS probes (AppNano) with tip radius of around 2 nm, were used to image the exposed cytoplasmic side of the membrane samples in tapping mode. Height, amplitude and phase images were captured at a resolution of 512 × 512 pixels for 8 µm×8 µm or 1 µm×1 µm areas and scan rate of 1 Hz.

All the AFM images were recorded using the software Nanoscope 5.1 (Veeco) or NanoWizard II (JPK Instruments AG), respectively.

### Analysis of AFM images

The images were processed and analyzed using software Nanoscope 5.31 (Veeco), NanoWizard Imaging Processing software (JPK Instruments AG), ImageJ 1.42q (Wayne Rasband) and Matlab R2009a (The Mathworks), and statistically studied using Graphpad Prism 5 (Software Mackiev) and Minitab 15 (State College).

### Skeletonization of the cytoskeleton

Ridge detection was used to skeletonize the cytoskeleton of uninfected and infected erythrocytes based on the AFM data (ASCII) using Matlab. Briefly, each pixel value was compared with the surrounding 8 pixel and those points which have less than 4 surrounding pixels with larger values were kept as the ridges, i.e. skeletonized spectrin network (Fig. S4). Isolated ridges (e.g. single pixels) within certain distance (e.g. two pixels) of no other connected ridges were removed as noise.

#### Measurement of the spectrin lengths

The spectrin lengths were measure as previously described [14]. Briefly, a line was drawn along the spectrin from one end (i.e. junction) to the other end and the length of the line was measured as the length of the spectrin (Fig. S5). Ten to seventeen images of cytoskeleton at each particular stage were randomly chosen and ten spectrin filaments were also randomly chosen and measured from non-knob areas from each of the chosen images.

#### Measurement of the mesh sizes

Method I: Studying the average mesh size of each skeletonized image data (one image represents one cell (C*_i_*_,_ i = 1, 2, …, n)). We assume that the boundaries of meshes re of very thin filaments (i.e. skeletonized spectrin proteins) and can be omitted in our calculation of the mesh size. So the sum of the mesh sizes of one image (C*_i_*) can be replaced by the scan size of the image. The number of the meshes (N*_m_*) can be obtained by calculating the numbers of the valleys (N*_v_*), which are the pixels with the smallest values locally obtained using Matlab and which represents the lowest points of each mesh (Fig. S4). Consequently, the average mesh size of one image (i.e. the mesh size of one cell at particular infection stage) (M*_i_*) can be obtained by using the quotient of the scan size of the image (S*_i_*) and the number of meshes (N*_mi_*), i.e. M*_i_*  = S*_i_*/N*_mi_*. Therefore, the average mesh size at one particular infection stage (M_a_) would be the average of all the M*_i_*, i.e. M_a_  = (M*_1_*+M*_2_*+…M*_i_*+…+M*_n_*)/n.

Method II: 100 meshes were randomly chosen from each image of each specific infection stage for statistical study. Loops were drawn along the boundaries of the meshes and the areas within the loops were measured as the mesh size using ImageJ (Fig. S6). To study the biggest mesh sizes of each image, ten images of each specific infection stage were randomly chosen for statistical study. The biggest ten meshes of each chosen image were measured and recorded using ImageJ.

#### Calculation of the normalized spectrin abundance (Csn)

We assume that each spectrin filament can be replaced by a line of ridges which has the same length of the represented spectrin protein but principally one-pixel wide. So the spectrin abundance (Cs) can be obtained by calculating the ratio of the number of the pixels representing the ridges to the total number of the pixels of certain image, which can be done by the bearing analysis using the Nanoscope software. The spectrin abundance values (Cs) were divided by the relative scan size (S) (e.g. 1.2 µm/1.0 µm = 1.2) to get the normalized spectrin abundance ratio (Csn), that is Csn = Cs/S. For the normalized spectrin abundance at knob area, circular areas with the diameter of 100 nm around each specific knob were analyzed (Fig. S7).

All values are reported as mean 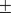 SD and n refers to the number of measurements or observations.

## Supporting Information

Figure S1 **Sorting cells by FACS.** (a) Frequency distributions of fluorescence values of Portion I & II. (b) Giemsa stained smears of each collected segment of FACS: the two sorted segments from Portion I, (PI-1) uninfected erythrocytes & (PI-2) rings; and the four sorted segments from Portion II, (PII-1) early trophozoites, (PII-2) mid trophozoites, (PII-3) late trophozoites and (PII-4) schizonts,. Bars, 10 µm.
(TIF)

Figure S2 **Histogram distribution and normal plots of the spectrin length at different stages of infection: (a) uninfected, (b) ring, (c) early trophozoite, (d) mid trophozoite, (e) late trophozoite and (f) schizont.**
(TIF)

Figure S3 **Histogram distribution and log-normal plots of the largest mesh sizes at different stages of infection using Method II: (a) ring, (b) early trophozoite, (c) mid trophozoite, (d) late trophozoite and (e) schizont and (F) the lognormal plots of the biggest mesh size at different stages of infection.**
(TIF)

Figure S4 **Measurement of the spectrin length by drawing lines along the spectrin from one end (junction) to the other end (junction).** The lines representing spectrin proteins were labeled with numbers. Bar scale, 500 nm.
(TIF)

Figure S5 **Illustration of the skeletonization for further statistical studies.** AFM data (512 × 512 pixels) were processed by ridge and valley detection using Matlab. The pixels which had less than 4 surrounding pixels with larger values were kept as the ridges. The pixels which had 8 surrounding pixels with larger values were kept as the valleys. The number of valley represented the number of the meshes in the representative image.
(TIF)

Figure S6 **Calculation of the largest meshes by drawing loops along the surrounding spectrins.** The areas within the loop labeled with numbers represented the size of the meshes. Bar scale, 500 nm.
(TIF)

Figure S7 **Calculation of the spectrin abundance at knob areas.** The areas which are 100 nm in diameter and have the same centre as the knobs were considered knob areas.
(TIF)
