# Supplementary figures and images for: Life Cycle-Dependent Cytoskeletal Modifications in Plasmodium falciparum Infected Erythrocytes
Source: PLoS One. 2013 Apr 9;8(4):e61170. doi: 10.1371/journal.pone.0061170 (PMC3621960; doi:10.1371/journal.pone.0061170)

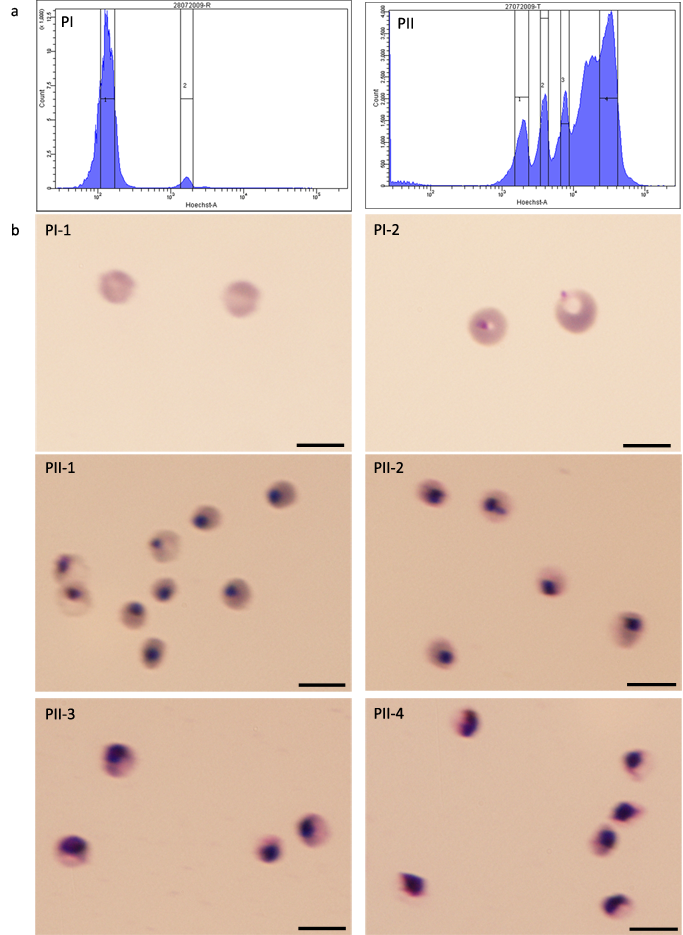

Supplement: Figure S1 — Sorting cells by FACS. (a) Frequency distributions of fluorescence values of Portion I & II. (b) Giemsa stained smears of each collected segment of FACS: the two sorted segments from Portion I, (PI-1) uninfected erythrocytes & (PI-2) rings; and the four sorted segments from Portion II, (PII-1) early trophozoites, (PII-2) mid trophozoites, (PII-3) late trophozoites and (PII-4) schizonts,. Bars, 10 µm. (TIF) [file pone.0061170.s001.tif]

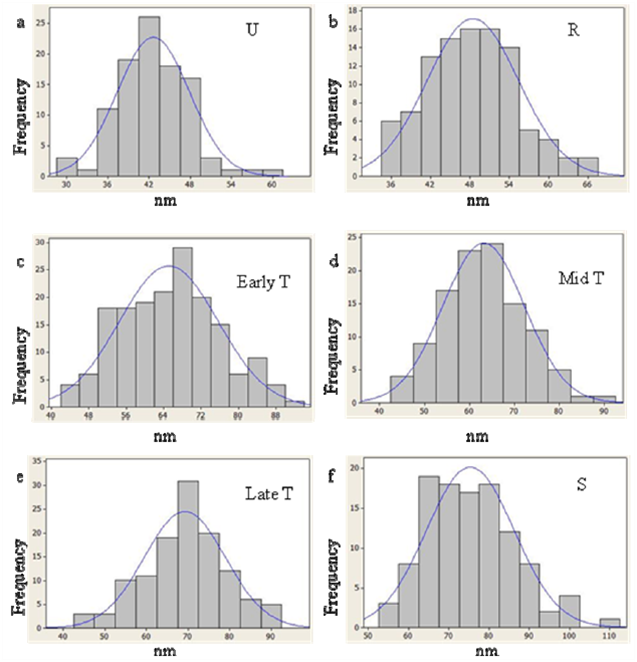

Supplement: Figure S2 — Histogram distribution and normal plots of the spectrin length at different stages of infection: (a) uninfected, (b) ring, (c) early trophozoite, (d) mid trophozoite, (e) late trophozoite and (f) schizont. (TIF) [file pone.0061170.s002.tif]

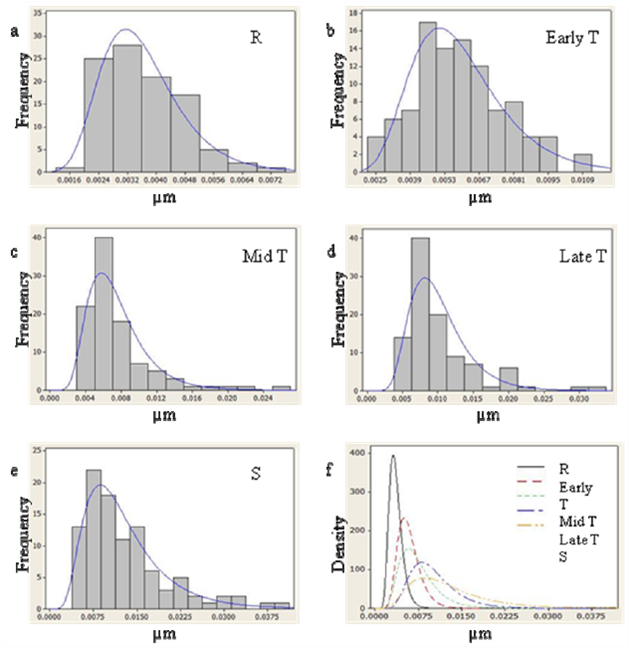

Supplement: Figure S3 — Histogram distribution and log-normal plots of the largest mesh sizes at different stages of infection using Method II: (a) ring, (b) early trophozoite, (c) mid trophozoite, (d) late trophozoite and (e) schizont and (F) the lognormal plots of the biggest mesh size at different stages of infection. (TIF) [file pone.0061170.s003.tif]

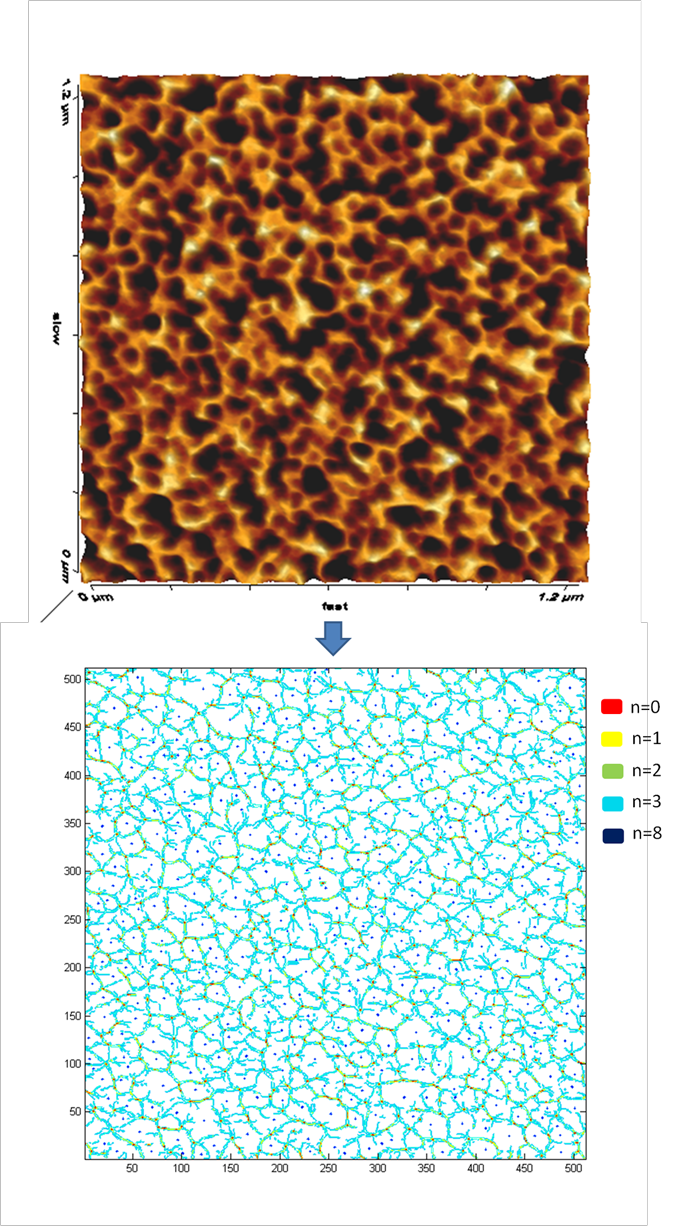

Supplement: Figure S4 — Measurement of the spectrin length by drawing lines along the spectrin from one end (junction) to the other end (junction). The lines representing spectrin proteins were labeled with numbers. Bar scale, 500 nm. (TIF) [file pone.0061170.s004.tif]

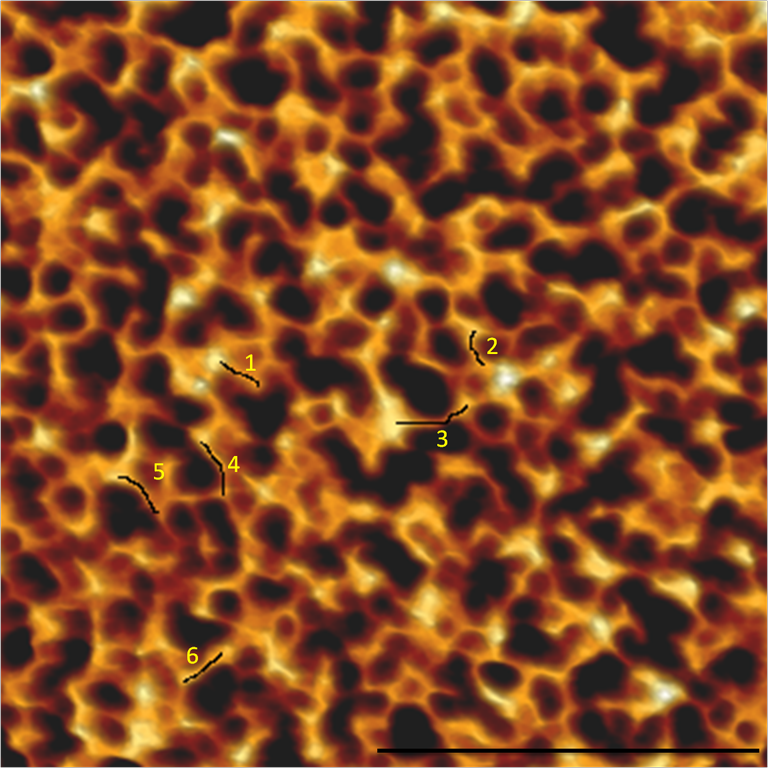

Supplement: Figure S5 — Illustration of the skeletonization for further statistical studies. AFM data (512 × 512 pixels) were processed by ridge and valley detection using Matlab. The pixels which had less than 4 surrounding pixels with larger values were kept as the ridges. The pixels which had 8 surrounding pixels with larger values were kept as the valleys. The number of valley represented the number of the meshes in the representative image. (TIF) [file pone.0061170.s005.tif]

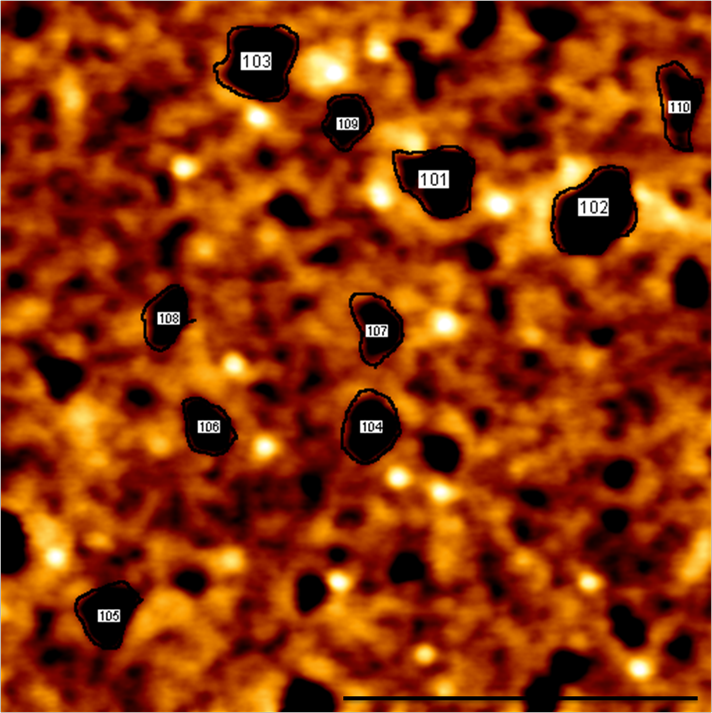

Supplement: Figure S6 — Calculation of the largest meshes by drawing loops along the surrounding spectrins. The areas within the loop labeled with numbers represented the size of the meshes. Bar scale, 500 nm. (TIF) [file pone.0061170.s006.tif]

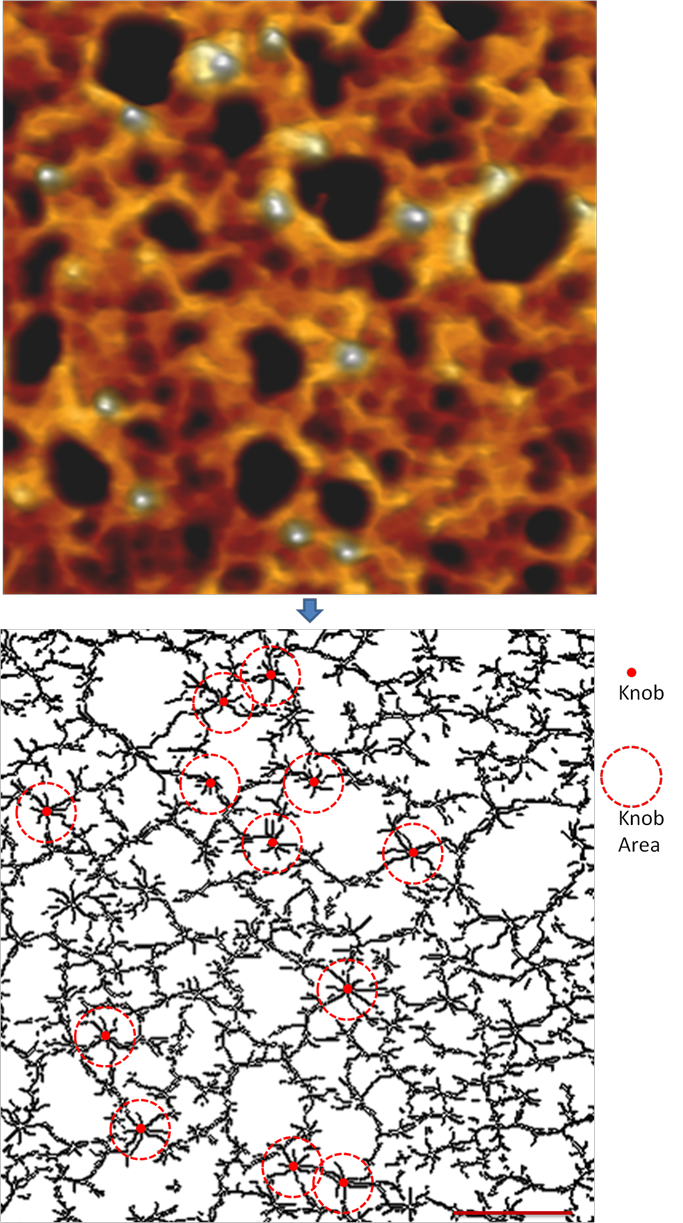

Supplement: Figure S7 — Calculation of the spectrin abundance at knob areas. The areas which are 100 nm in diameter and have the same centre as the knobs were considered knob areas. (TIF) [file pone.0061170.s007.tif]
